# Evaluation of a Microbial Inhibitor in Artificial Diets of a Generalist Caterpillar, *Heliothis virescens*

**DOI:** 10.1673/031.010.19701

**Published:** 2010-11-12

**Authors:** Karl A. Roeder, Indira Kuriachan, S. Bradleigh Vinson, Spencer T. Behmer

**Affiliations:** Department of Entomology, Texas A&M University, College Station, TX, 77843-2475, USA

**Keywords:** fungus, insect rearing, mold, nutrition, physiology

## Abstract

Controlling microbial growth in artificial diets is a key component in the rearing of laboratory insects. In this study an antimicrobial agent, Diet Antimicrobial Agent (DAA), was tested for its ability to suppress microbial growth on a range of different diets, and for its effect on larval and pupal performance of individuals from two different strains of *Heliothis virescens* Fabricus (Lepidoptera: Noctuidae). In the first experiment, it was found that the presence of DAA in a pinto bean-based diet was highly effective at suppressing microbial growth relative to other methods, and that survival of caterpillars on diets with DAA was superior to other treatments. Caterpillars also performed best on diets with DAA, although this may have been the result of laboratory selection pressure as these caterpillars had been reared on pinto bean-based diets with DAA for several hundred generations. A second experiment was conducted, using different diets and a different strain of *H. virescens* to more fully evaluate DAA. Here it was found that DAA significantly suppressed microbial growth and development, particularly in synthetic diets. There was no significant effect of DAA on pupal development time or mass gain. There was a statistically significant effect of DAA on eclosion time for two of the diets, although the effect did not seem to be biologically meaningful. The findings suggest that DAA is an effective suppressor of microbial growth on artificial diets, and that its net effect on developing diet-reared insects is neutral.

## Introduction

Insects reared in the laboratory on artificial diets are used in a number of different fields ranging from biological control (Knipling 1979; Bellows et al. 1999) to food for humans (Bodenheimer 1951; Defoliart 1999). However, contamination of insect artificial diets with microbial growth, particularly mold and fungus, is a common problem. Control of microbial agents is essential for maintaining healthy insect colonies and for the assurance of meaningful studies (Cohen 2003).

Generally there are two broad classes of compounds used to control microbial agents that can contaminate insect artificial diets: antibacterial and antifungal. However, many of these can be quite toxic to insects, even at low concentrations (Cohen 2003). For example, methyl paraben, a common antifungal agent, significantly reduces an insect colony's biomass as its concentration in the diet increases (Alverson and Cohen 2002). Finding the right combination of compounds to fight microbial growth, while at the same time limiting any negative effects these antimicrobial compounds have on the insects being cultured, can be quite challenging.

The aim in this study was to investigate the efficacy of a microbial inhibitor that had been developed through repeated trial and error experiments to rear *Heliothis virescens* Fabricus in the Vinson lab over the past 15 years. This inhibitor, henceforth called diet antimicrobial agent (DAA), was tested using two strains of tobacco budworm, *Heliothis virescens*, on a range of artificial diets, and across a range of different microbial control strategies, to determine its efficacy against microbial contamination and its effect on caterpillar performance. The results demonstrate that DAA gives excellent microbial control, and at the concentrations used in the current experiment has no negative effects on caterpillar performance. The results also demonstrate that DAA works across different strains of caterpillars.

## Materials and Methods

This study was divided into two experiments. The first used caterpillars from a culture of *H. virescens* maintained at Texas A&M University. Here microbial growth and insect performance on a single diet across five different microbial management strategies was measured, including the DAA treatment. The second experiment used *H. virescens* from a culture maintained at North Carolina State University, and investigated the effectiveness of DAA across three different diets.

### Diet Antimicrobial Agent

DAA is composed of five key ingredients (Table 1), based on their ability to control microbial growth. The first and most abundant compound is propionic acid, a carboxylic acid that inhibits mold and some bacteria growth by lowering the pH of the diet. The second compound is phosphoric acid, a sour tasting inorganic acid that is used in the acidification of food and beverages, and as a nutrient buffer in antibiotic manufacture (Kirk and Othmer 1978a). The third compound is sorbic acid, a hexadienoic acid that successfully controls bacteria, molds and yeasts in moist foods that have a pH below 6.5 (Kirk and Othmer 1978b). The fourth compound is benzoic acid, an aromatic carboxylic acid that is effective at controlling microbial growth at pH levels between 2.5–4.5; at low concentrations (e.g. 0.1%) it effectively prevents bacterial growth (Kirk and Othmer 1978c). The last compound is chloramphenicol, an antibiotic used to stop bacteria both gram-positive and gram-negative through the inhibition of bacterial protein synthesis.

### Experiment One

**Experimental insects.** Caterpillars of *H. virescens* came from a culture at Texas A&M University where they were reared on a pinto bean diet (Table 2) that was commercially available and prepared according to the directions from Bioserv (Bioserv #F9394B).

**Treatments.** In total there were five treatments with each treatment containing the pinto bean based diet described above. The treatments used were;

1. Standard pinto bean diet (PB) with no modifications.
2. Standard PB diet replaced every 3 days. (PB[3])
3. PB diet + 0.1 ml of formalin per 40 grams of dry diet. (PB-F)
4. PB diet + 0.1 ml of formalin per 40 grams of dry diet, replaced every 3 days. (PB-F[3])
5. PB diet + 0.1 ml of formalin and 0.5 ml DAA per 40 grams of dry diet. (PB-FI)

The diets were prepared in large quantities and suspended in a 1% agar solution with a 4:1 agar solution:dry ingredient ratio. The microbial inhibitor products (formalin, e.g. 40% aqueous solution of formaldehyde and/or DAA) were added to wet diet mixtures while it was still in liquid form. The diet and microbial inhibitor products were mixed thoroughly with the agar diet solution and then set aside to cool and solidify.

**Table 1.**
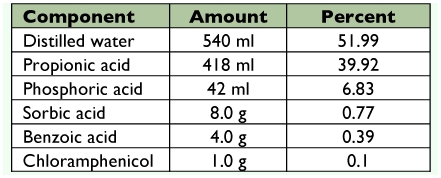
Diet Antimicrobial Agent (DAA) ingredient list, expressed in total amounts and as a percent.

**Table 2.**
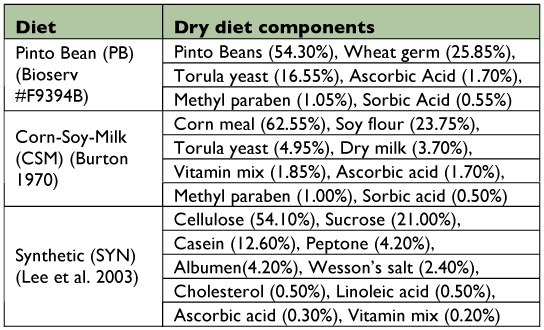
Dry components, expressed as a percent of the total, for each diet treatment.

**Protocol 1.** Twenty-five caterpillars were randomly assigned to each of five treatments, and larvae were placed individually on a slice of diet that weighed approximately three grams. These diet blocks were housed in cylindrical plastic vials, which measured 6.5 ×× 2 cm, and a small cylindrical sponge closed the open end of each vial. The experimental incubator used was set at 29°° C +/- 1°°C with a 14:10 L:D. Four separate variables were recorded for each vial during the course of this experiment: (1) microbial development, recorded as either present or absent; (2) larval survival, recorded as individuals successfully pupating; (3) developmental time, recorded as the number of days from placement of neonate on food to pupation and (4) pupal mass, recorded in mg, approximately 4 days after pupation.

### Experiment Two

**Experimental insects.** Caterpillars used in this experiment came from a culture at North Carolina State University that had been reared on a corn-soy-milk based diet (Table 2), modified from Burton (1970).

**Treatments.** This experiment used three diets each with DAA either present or absent yielding six unique treatments. The first diet treatment was a modified Corn-Soy-Milk (CSM) diet from Burton (1970). The second diet was a synthetic diet (SYN) as described in Simpson and Abisgold (1985), modified from Dadd (1961), with sucrose as the primary digestible carbohydrate (Table 2) (Lee et al. 2003). It had a total macronutrient content of 42%, with protein (P) and digestible carbohydrate (C) each present at equal amounts. The last diet was a combination of the first two diets (CSM + SYM). This diet was 20% CSM and 80% SYN, with the total macronutrient content and P:C ratio equal to those found in the SYN diet.

All diets were prepared in 200 ml quantities and presented to the insects in a 1% agar solution with a 4:1 agar solution:dry ingredient ratio. Formalin and DAA were added to wet diet mixtures after the combination of dry and agar components had been completed, but while the diets were still in liquid form. Formalin was added at 0.1 ml/40 g of dry diet, while DAA was added at 0.5 ml/40 g of dry diet.

**Protocol 2.** Initially, thirty 2 oz. Solo®® cup arenas for each of the six treatments were set aside to measure the effectiveness of DAA on retarding microbial growth in the absence of caterpillars, for a period of 14 days. Each arena was observed daily, noting the presence or absence of microbial growth. Photographs of each arena were taken on the third and seventh days, using a Nikon Coolpix L12.

The second part of experiment two examined caterpillar growth and development on the three diets in the presence and absence of DAA. Our aim here was to determine the direct effect (positive, negative or neutral) of DAA on caterpillar performance. Twenty-five individuals were assigned to each of six treatments. Approximately 1 g blocks of diet were initially placed in clean 2 oz. Solo®® cup arenas and changed every three days. Caterpillar survival to the pupal stage and eclosion to the adult stage was recorded for each individual. For those caterpillars that molted and eclosed, the time of these events was recorded in days. Pupal mass was also recorded (in mg) four days after entering the pupal stage. If microbial growth was observed in an arena prior to the scheduled food change, the occupying caterpillar was transferred to a new container and given a fresh block of food.

**Statistical analysis.** Analyses were performed using JMP 7.0.2 and SAS 9.2.1 (SAS Institute Inc). Logistic regression tests were used to compare survival success to the pupal and adult stages. When significant effects were observed, odds ratios were employed to make comparisons between treatments. Survival Analyses were run to compare developmental time to pupation and eclosion, and where significant differences were detected contrasts were used to determine differences between the treatments (Fox 1993). One-way ANOVA was used to compare pupal mass, and a Student's t-test was employed for treatment comparisons.

## Results

### Experiment one

**Figure 1.**
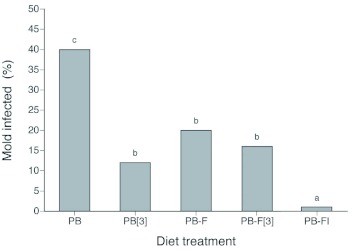
Diets infected with microbial agents expressed as a percent of the total number of replicates for each treatment. Diets used were PB (pinto bean), PB-FI (Pinto bean with formalin and DAA), PB-F (Pinto bean with formalin), PB[3] (Pinto bean that was changed every three days), and PB-F[3] (Pinto bean with formalin that was changed every three days). Letters above the bars indicate significant differences between the treatments. High quality figures are available online.

**The appearance of microbial growth on the different treatments.** There was a significant difference among the treatments in terms of microbial appearance (Logistic regression: df = 4, χ?^2^ = 17.32, *P* = 0.002; Figure 1). Approximately 40% of the vials holding PB diet contained microbial growth, and the addition of formalin to the diet significantly reduced the occurrence of microbes (down to approximately 20% of the vials). Changing the food every three days (e.g. PB[3] and PB-F[3]) did not significantly reduce the occurrence of microbial growth, relative to the PB-F treatment. The best control of microbial growth was observed on the PB-FI treatment, which contained DAA. Here microbes were only observed in a single vial, and this level of occurrence was a significant improvement over the PB-F, PB[3] and PB-F[3] treatments.

**Figure 2.**
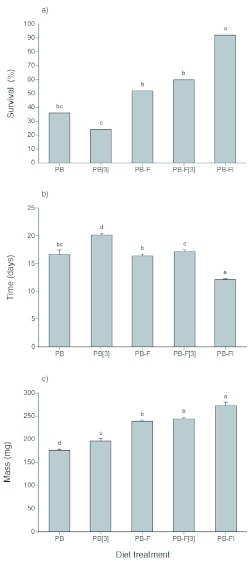
Performance of *Heliothis virescens* caterpillars on different regimes for managing microbial growth on a pinto bean-based diet. Panel (a) shows survival percent from hatching to pupation, (b) shows mean development time (±± SE) from hatching to pupation and (c) shows mean final mass (±± SE) of caterpillars that successfully pupated. Diets used were PB (pinto bean), PB-FI (Pinto bean with formalin and DAA), PB-F (Pinto bean with formalin), PB[3] (Pinto bean that was changed every three days) and PB-F[3] (Pinto bean with formalin that was changed every three days). Letters above the bars indicate significant differences between the treatments. High quality figures are available online.

**Affect of different treatments on insect performance.** Survival to pupation was significantly different among the treatments (Logistic regression: df = 4, χ?^2^ = 30.46, *P* < 0.001). As shown in figure 2a, it was highest on the PB-FI treatment (> 90%) and lowest on the treatment lacking any anti-microbial agents (PB and PB[3]). The addition of formalin improved survival relative to the PB[3] treatment, but not compared to the PB treatment.

Two variables were measured with respect to performance: developmental time (recorded in days from hatch to pupal stage) and mass gain. Development time was significantly affected by the treatment (Survival analysis: df = 4, χ?^2^ = 149.51, *P* < 0.001), as was pupal mass (ANOVA: df = 4, F = 53.71, *P* < 0.001). Development was fastest on the PB-FI treatment, slowest on the PB[3] treatment and intermediate on the other three treatments (Figure 2b). In terms of growth, measured as mass gain, caterpillars were largest on the PB-FI treatments and smallest on the PB treatment (Figure 2c). Changing the PB diets every 3 days improved mass gain relative to the PB treatment and addition of formalin (PB-F and PB-F[3]) improved mass gain even more. However, these latter two treatments were still inferior to the PB-FI treatment.

### Experiment two

**Figure 3.**
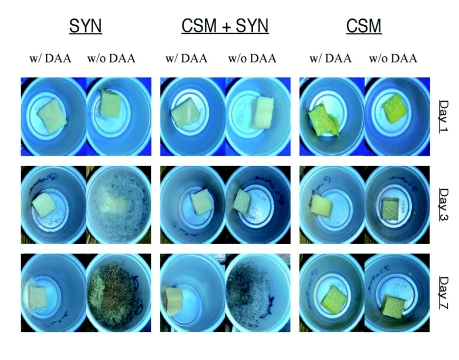
Photographs of diet blocks from experiment two showing the amount of microbial growth and development over a seven-day period (in the absence of caterpillars). Three different diets (CSM, CSM + SYN and SYN), with or without Diet Antimicrobial Agent (DAA) are shown, across three different days. High quality figures are available online.

**The appearance of microbial growth on the different treatments.** When caterpillars were absent from the CSM diet blocks (with or without DAA) no microbial growth was observed on the diet blocks after 14 days. In contrast, microbial growth was observed on the other two diets (SYN and CSM + SYM). On these two diets the addition of DAA significantly delayed the appearance of microbial growth (Table 3). On average, microbes appeared three times faster on the SYN and CSM + SYN diets lacking DAA (Table 3). Figure 3 shows digital photographs of the overall size and robustness of the developing microbial growth over a weeklong time period for diets with and without DAA.

**Figure 4.**
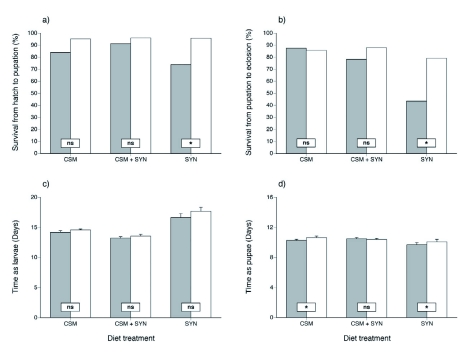
Survival success and developmental time of *Heliothis virescens* for experiment two, across three different diets (CSM, CSM + SYN, and SYN) with or without Diet Antimicrobial Agent (DAA). Grey columns represent diets without DAA while white columns represent diets with DAA. Panels (a) and (b) show survival expressed as a percent of the total replicates for larva and pupa, respectively. Panels (c) and (d) show mean development time (±± SE) for larva and pupa, respectively. Rectangular boxes on the different diets reveal whether there are significant differences between similar diets, with and without DAA (ns = not significant, ** = *P* < 0.05). High quality figures are available online.

**Affect of DAA on insect performance.** Survival to pupation and pupation to eclosion, was analyzed separately for each diet (CSM, CSM + SYN, and SYN). Survival to pupation was always better on diets containing DAA, but only on the SYN diets was this difference significant (Table 4; Figure 4a). The trends for survival to eclosion were more mixed, but again only on the SYN treatment was a significant difference observed with eclosion success being significantly better on SYN diets containing DAA (Table 4; Figure 4b).

**Figure 5.**
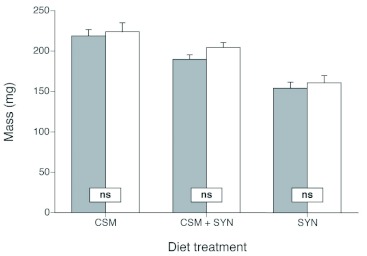
Final pupal mass (±± SE) of *Heliothis virescens* across three different diets (CSM, CSM + SYN and SYN), with or without Diet Antimicrobial Agent (DAA). Grey columns represent diets without DAA while white columns represent diets with DAA. Rectangular boxes on the different diets reveal whether there are significant differences between similar diets, with and without DAA (ns = not significant, ** = *P* < 0.05). High quality figures are available online.

With respect to performance, the addition of DAA to the diets did not significantly affect development time from the hatch to pupation (Table 4; Figure 4c). We did, however, detect differences in time from pupation to eclosion differences in time from pupation to eclosion on two of the three diets (Table 4; Figure 4d). Eclosion time was significantly delayed when DAA was in the CSM and SYN diets. With respect to mass gain, no significant differences were observed for any of the three diets (Table 4; Figure 5).

### Discussion

The prevention or delay of microbial growth (e.g. mold and fungus) is a key component in the successful rearing of insects. Artificial diets are a rich and pure source of nutrients and microbial growth, when not properly controlled, can opportunistically exploit and grow on confined artificial diets (Cohen 2003). In fact, Inglis and Cohen (2004) found that diets lacking antibacterial or antifungal agents supported a full complement of fungi and bacteria within 24 hours. Diet manipulations through the use of antimicrobial agents can have profound effects on insects in a multitude of different ways. Our results show, however, that DAA significantly suppresses microbial growth on a range of artificial diets and that aside from increasing caterpillar survival, its net effect on insect performance is neutral.

**Table 3.**
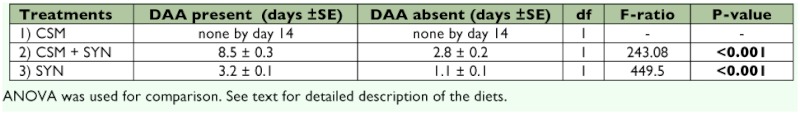
Mean (±±SE) number of days until microbial agents appear on different diets, in the absence of caterpillars.

**Table 4.**
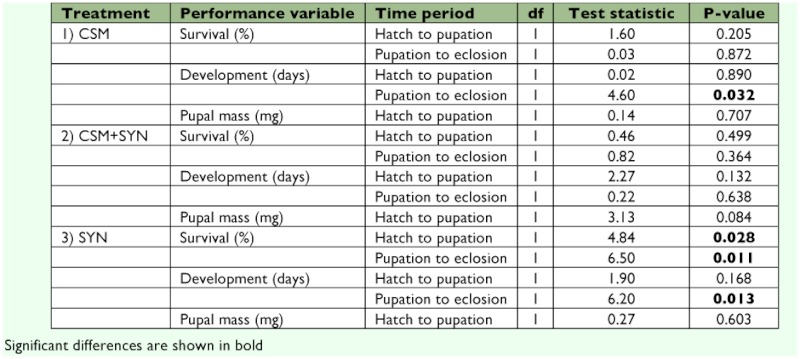
Statistical values for differences when DAA is present or absent using Logistic Regressions (survival), Survival Analyses (development time), and one-way ANOVA (pupal mass) in experiment two, part two.

Different strategies can be employed to control microbial growth on insect artificial diets. In the current study microbes grew quite readily when antifungal agents were absent —— over 40% of the PB replicates showed microbial growth and development over a two to three week time period. Attempts to control microbial growth by changing foods regularly (every 3 days) did result in a decrease in contamination, but effects on caterpillar performance were mixed and in the case of development this strategy had a negative effect. The addition of formalin, e.g. 40% aqueous solution of formaldehyde, is a common strategy used to control microbial growth in many insect diets, and when it is added to the PB diet mold development is significantly decreased by 20%. Many insects can tolerate formalin at low concentrations, but there is concern about its direct negative effect on insect performance. Formaldehyde, the major component of formalin, is a simple aldehyde that is used in a number of different fields, for example in textiles and as disinfectants (NICNAS 1997), but it has also been classified as a probable human carcinogen by the U.S. Environmental Protection Agency and a cause of nasopharyngeal cancer in humans by the International Agency for Research on Cancer. The Texas A&M *H. virescens* culture has, however, been reared on diets containing a low concentration of formaldehyde for several hundred generations, so it is possible that this strain has been selected to perform well on diets containing formaldehyde.

The addition of DAA to PB diet that contained formalin greatly suppressed microbial growth, so the two antimicrobial agents appear to be synergistic. Insect performance compared to the other diets also increased when DAA was present. However, this could be due to adaptation of the Texas A&M *H. virescens* colony to a diet containing DAA for several hundred generations.

To overcome potential historical effects of DAA on *H. virescens* performance (Bartlett 1984, 1994), a second set of *H. virescens* caterpillars were obtained from North Carolina State University (NCSU). Here the NCSU caterpillars were tested on one of three different diets, ranging from plant-based to synthetic-containing (i.e. using chemicals off the shelf such as casein and sucrose) with or without DAA. First, however, the effects of DAA on suppressing microbial growth in the absence of caterpillars were examined. Interestingly the CSM diet, on which the NCSU *H. virescens* culture is typically reared, had no visibly detectable microbial growth after 14 days. The CSM diet recipe contains methyl paraben, a well-known and commonly used antifungal agent (Cohen 2003), and its presence may have been instrumental in suppressing microbial growth. On the other hand, microbial growth was observed on the CSM + SYN treatments, even though it too contained methyl paraben, although at a reduced concentration compared to the CSM diet. On the CSM + SYN diets the addition of DAA clearly suppressed microbial growth, as its appearance was delayed 8.5 days, compared to 2.8 days when DAA was absent. The data also suggest that pure synthetic diets, even ones that contain a small amount of formaldehyde (e.g. SYN), are highly susceptible to microbial growth (typically within 1 day). On these diets the addition of DAA is clearly beneficial, because microbial growth was, on average, first observed after 3 days.

Having shown the effectiveness of DAA in suppressing microbial growth, the effect DAA had on caterpillar performance still needed to be determined. DAA was found to have no significant positive or negative effect on larval development for the three diets tested (CSM, CSM + SYN, and SYN), and there was no positive or negative effect on mass gain even though there was an observed trend in which growth on the diets (in terms of mass gain) decreased as the proportion of SYN in the diet increased. More importantly, the observed significant time to eclosion effect of DAA on the CSM and SYN diets was very small (on average ¼¼ of a day). Although these results are statistical effects, we do not feel they are biologically meaningful. These results, when combined with the increased survival rates observed on diets containing DAA, make a strong case for the more widespread use of DAA in insect diets as an effective tool for suppressing microbial growth without concern for toxic effects on the insects themselves.

## Abbreviation

DAA,: diet antimicrobial agent
